# Enzyme kinetics from circular dichroism of insulin reveals mechanistic insights into the regulation of insulin-degrading enzyme

**DOI:** 10.1042/BSR20181416

**Published:** 2018-11-07

**Authors:** Valerie A. Ivancic, Claire A. Krasinski, Qiuchen Zheng, Rebecca J. Meservier, Donald E. Spratt, Noel D. Lazo

**Affiliations:** Carlson School of Chemistry and Biochemistry, Clark University, 950 Main Street, Worcester, MA 01610, U.S.A.

**Keywords:** circular dichroism, insulin-degrading enzyme, insulin, Michaelis-Menten kinetics

## Abstract

Insulin-degrading enzyme (IDE) is a zinc metalloprotease that selectively degrades biologically important substrates associated with type 2 diabetes and Alzheimer’s disease (AD). As such, IDE is an attractive target for therapeutic innovations. A major requirement is an understanding of how other molecules present in cells regulate the activity of the enzyme toward insulin, IDE’s most important physiologically relevant substrate. Previous kinetic studies of the IDE-dependent degradation of insulin in the presence of potential regulators have used iodinated insulin, a chemical modification that has been shown to alter the biological and biochemical properties of insulin. Here, we present a novel kinetic assay that takes advantage of the loss of helical circular dichroic signals of insulin with IDE-dependent degradation. As proof of concept, the resulting Michaelis–Menten kinetic constants accurately predict the known regulation of IDE by adenosine triphosphate (ATP). Intriguingly, we found that when Mg^2+^ is present with ATP, the regulation is abolished. The implication of this result for the development of preventative and therapeutic strategies for AD is discussed. We anticipate that the new assay presented here will lead to the identification of other small molecules that regulate the activity of IDE toward insulin.

## Introduction

**Figure 1:**
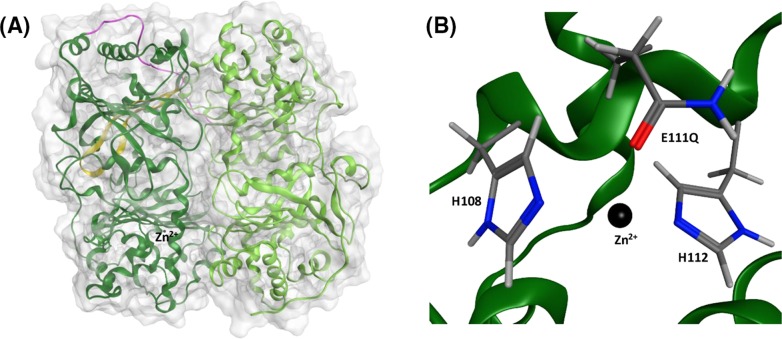
Structure of IDE determined by X-ray crystallography (PDB ID 4PES [7]) (**A**) Molecular surface of IDE along with the ribbon representation of its tertiary structure. IDE is composed of an N-terminal half (IDE-N, dark green) and a C-terminal half (IDE-C, light green) linked by a flexible linker (magenta). IDE-N contains a conserved exosite (yellow) which contains an anti-parallel β-sheet. (**B**) The active site is found in IDE-N and contains the HXXEH motif, in which the two histidines (H108 and H112) coordinate Zn^2+^ and the glutamate (E111 mutated to a glutamine in 4PES) participates in the hydrolysis of peptide bonds.

Insulin-degrading enzyme (IDE) is an attractive target [1,2] for the development of novel therapeutic strategies for type 2 diabetes (T2D) and Alzheimer’s disease (AD), because it degrades amylin [1], which self-assembles to form assemblies that are toxic to the insulin-producing pancreatic β-cells [3] and the amyloid-β protein (Aβ) [2,4], which aggregates to form neurotoxic oligomers [5]. IDE is composed of an N-terminal half (IDE-N) and a C-terminal half (IDE-C) joined by a flexible loop (PDB ID 4PES, Figure 1A) [6,7]. The two halves come together to form a catalytic chamber, also known as a crypt, with a volume of approximately 16,000 Å^3^, excluding substrates that contain more than 80 amino acids [6,8–12]. The crypt of IDE also has interesting electrostatic properties in that the predominantly negative interior of IDE-N complements the predominantly positive interior of IDE-C. IDE-N contains a highly conserved exosite (Figure 1A), which has been hypothesized to anchor IDE’s substrates prior to degradation [1,8], and the active site (∼30 Å away from the exosite) which contains the Zn^2+^ binding motif HXXEH (Figure 1B), in which the two histidines (H108 and H112) coordinate Zn^2+^ and the glutamate residue (E111, replaced by glutamine in 4PES) is directly involved in the hydrolysis of peptide bonds. Replacing E111 with glutamine deactivates IDE [13].

The ability of IDE to degrade amyloidogenic substrates suggests that the development of small-molecule activators that increase its activity is an attractive therapeutic strategy for T2D and AD. However, enhancing the activity of IDE has deleterious consequences. As its name implies, IDE also degrades insulin [14,15], a hormone that regulates blood glucose levels by helping cells take in glucose. A more attractive therapeutic strategy, therefore, is one that will enhance the IDE-dependent clearance of the amyloidogenic substrates without offsetting the normal levels of insulin. An important requirement of this strategy is the identification of regulators that govern the activity of the enzyme toward insulin *in vivo*.

Insulin is composed of two chains (A and B), joined together by two interchain disulfide bonds (Cys^A7^−Cys^B7^ and Cys^A20^−Cys^B19^). Previous kinetic studies on the identification of potential regulators of the IDE-dependent degradation of insulin have used the trichloroacetic (TCA) assay [16,17], presumably because of the impracticality of using HPLC for insulin degradation assays, as discussed by Duckworth [18]. The TCA assay, however, requires monoiodination of one of four tyrosines (Tyr^A14^, Tyr^A19^, Tyr^B16^, and Tyr^B26^) of insulin, and the location of iodine can affect the kinetics of the degradation [18]. The latter is to be expected, since experimental and theoretical studies have shown that site-specific iodination of insulin modifies its biological and biochemical properties [19]. Quantum mechanical calculations have shown that the positive σ-hole of large halogens including iodine can form stabilizing interactions with proximate water molecules and polar groups [20–22]. Site-specific iodination of Tyr^B26^ enhances the binding of insulin to its receptor [23,24] and increases the resistance of a rapid acting insulin analog to fibrillation [25]. We hypothesize that the site-specific iodination of insulin also enhances the interaction of the protein with IDE, thus making the relevance of *in vitro* studies that used iodinated insulin to the degradation of insulin *in vivo* difficult to ascertain.

In the present study, we used circular dichroism (CD) to monitor the IDE-dependent degradation of unlabeled insulin. By using the observed ellipticity at 222 nm as a measure of the extent of degradation, we were able to obtain Michaelis–Menten kinetic constants in the absence and presence of adenosine triphosphate (ATP), which has been hypothesized to regulate IDE *in vivo* [10,16,17,26,27]. Our results show that ATP regulates IDE-dependent degradation of insulin, but the addition of Mg^2+^ abolishes the regulation. Importantly, this finding has implications for the development of therapeutic and/or preventative strategies for AD.

## Materials and methods

### Insulin-degrading enzyme expression and purification

The vector (pGEX-6p-1) encoding glutathione S-transferase tagged human insulin-degrading enzyme (GST-IDE) was kindly provided by Dr Malcolm A. Leissring (University of California, Irvine). The E111Q mutation was introduced into the vector using site-directed mutagenesis and verified by DNA sequencing. Wild-type and E111Q GST-IDE were expressed in *Escherichia coli* BL21 (DE3) codon plus competent cells and grown in LB broth at 37°C to an OD_600_ of 0.4. Expression was then induced with 50 µM isopropyl-β-1-thiogalactopyranoside, followed by incubation for 16 h at 200 rpm and 25°C. The cells were harvested by centrifuging at 1500×*g* for 10 min at 4°C. The pellet was then resuspended in 20 ml PBS with 400 µL of a 100-mM solution of the non-metalloprotease inhibitor phenylmethylsulfonyl fluoride in ethanol. The solution was then homogenized using an Avestin homogenizer to break open the cells, then centrifuged at 103,000×*g* for 40 min to separate the cell debris from the soluble protein. The IDE-containing supernatant was syringe filtered with a 0.2 µm filter and purified using a 5 mL GST Trap Fast Flow column (GE) on an ÄKTA Pure FPLC and eluted with PBS containing 10 mM glutathione. Fractions containing IDE were combined and incubated for 1 h at room temperature with 600 µL of GST PreScission protease (2.5 mg/mL) to cleave the GST-tag. The solution was dialyzed overnight in PBS at 4°C with gentle stirring. Purification by size-exclusion chromatography was completed using a HiLoad 16/600 Superdex 200 pg column, also connected to the ÄKTA Pure FPLC. The concentration of the purified protein was determined using UV absorbance at 280 nm (*ε*_280 nm_ = 113,570 M^−1^ cm^−1^ [28]). Glycerol was then added to the solution of IDE to 1% (v/v). After aliquoting, the solutions were flash frozen with liquid nitrogen and stored at −80°C.

### Preparation of stock solutions

Human insulin and ATP were purchased from Sigma–Aldrich (St. Louis, MO), and stock solutions of each molecule were prepared in 50 mM Tris buffer (pH 7.4). To overcome the low solubility of insulin at pH 7.4, the solutions were incubated at 37°C overnight, cooled down to room temperature the following day and then centrifuged at 16000×*g* for 1 min to remove undissolved protein. Concentrations were determined by UV absorbance at 276 nm (*ε*_276 nm_ = 6190 M^−1^ cm^−1^ [29]) for insulin and by UV absorbance at 259 nm (*ε*_259 nm_ = 15,400 M^−1^ cm^−1^ [30]) for ATP. MgCl_2_ was purchased from Fisher Scientific (Fair Lawn, NJ). Stock solutions of MgCl_2_ at 50 mM were prepared in 50 mM Tris buffer (pH 7.4).

### CD spectroscopy

Far UV CD spectra were recorded at 37°C using a JASCO J-815 spectropolarimeter. Quartz cuvettes with a path length of 1 mm were used. Each spectrum reported in this work was an average of four scans, with each scan recorded from 260 to 198 nm using 1 nm steps and an averaging time of 1 s. All samples were kept at 37°C in between recording of spectra.

### LC/MS

IDE-dependent digestions of insulin were conducted at 37 and at 4°C at a substrate-to-enzyme molar ratio of 100:1. Aliquots (18 µL) of each digestion were removed periodically and acidified with 8 µL of 1% (v/v) trifluoroacetic acid in water to quench the reactions. To break the disulfide bonds prior to LC/MS, the samples were neutralized and reduced immediately with 10 µL of 45 mM dithiothreitol for 30 min at 50°C. The samples were then alkylated in the dark with 10 µL of 100 mM iodoacetamide for 30 min at room temperature.

All LC/MS experiments were conducted at the Proteomics and Mass Spectrometry Facility of the University of Massachusetts Medical School. After drying the samples, they were diluted to 5 µM in a solution of 5% acetonitrile and 0.1% TFA. Peptide separation and identification by mass spectrometry were achieved using a NanoAcquity UPLC system (Waters Corporation) interfaced to an Orbitrap Q Exactive hybrid mass spectrometer (Thermo Fisher Scientific). The digests were fractionated using an analytical column packed with 25 cm of 3 µm Magic C18AQ (Bruker–Michrom) particles. Solvents A and B were 0.1% formic acid in water and 0.1% formic acid in acetonitrile, respectively. The elution program included a linear gradient developed from 5% solvent A to 35% solvent B in 45 min. Ions were introduced by positive electrospray ionization through a liquid junction into the mass spectrometer. Mass spectra were recorded from 300 to 1750 (m/z) at 70,000 resolution (m/z 200). Data-dependent acquisition chose the ten most abundant precursor ions for tandem mass spectrometry by higher energy collisional dissociation using an isolation width of 1.6 Da, collision energy of 27, and a resolution of 17,500.

Raw data files were processed using version 2.1 of Proteome Discoverer (Thermo Fisher Scientific) prior to database searching with version 2.5 of Mascot Server against the *Uniprot_Human* database. Search parameters did not include enzyme specificity. Variable modifications were considered, including N-terminal acetylation of the protein, oxidized methionine and pyroglutamic acid for N-terminal glutamine. Search results were then loaded into Scaffold Viewer (Proteome Software, Inc.).

### Enzyme kinetics

Seven insulin solutions in 50 mM Tris buffer (pH 7.4), each with a volume of 200 μL, were prepared in Eppendorf tubes. The concentration of the solutions ranged from 15 to 110 µM (15, 20, 25, 30, 50, 80, and 110 µM). The reaction was initiated with the addition of IDE at a concentration of 1 µM. The solution was then transferred into a 1-mm path length quartz cuvette which was then loaded into the sample holder of our CD spectrometer, where the ellipticity at 222 nm ([*θ*_obs(222 nm)_]) was recorded for 5 min at 37°C. The real-time [*θ*_obs(222 nm)_] data were then used to calculate the amount of digested insulin ([DI]) using (eqn 1). Initial rates (*V*_0_) were determined from plots of [DI] against time. Michaelis–Menten, Lineweaver–Burk, and Hanes–Woolf plots were constructed and used to determine the kinetic constants *K*_M_, *V*_max_, *k*_cat_, and *k*_cat_/*K*_M_.

## Results and discussion

**Figure 2:**
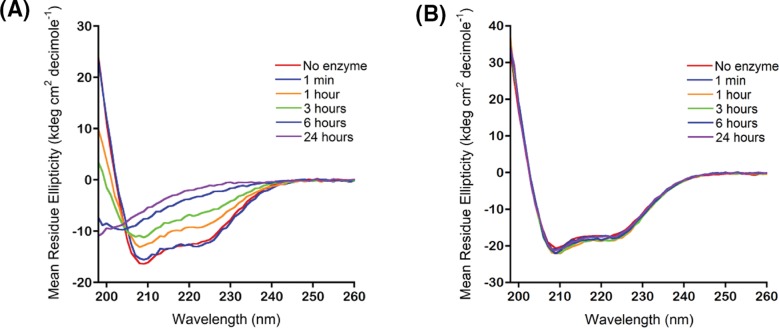
The far UV CD spectrum of insulin is sensitive to the extent of IDE-dependent degradation (**A**) In the presence of IDE, the helical dichroic signals of insulin at 222 and 208 nm are lost as digestion time is increased from 1 min to 24 h. (**B**) In the presence of E111Q IDE, the helical dichroic signals of insulin persisted. All spectra in (**A**) and (**B**) were collected at 37°C on 20 μM insulin in 50 mM Tris buffer, pH 7.4. The substrate-to-enzyme molar ratio was 100:1.

Our premise for using CD in our studies of the IDE-dependent degradation of insulin is based on the current working model of the IDE-dependent degradation of insulin [1,8]: the predominantly α-helical structure of insulin must unfold following its binding and degradation within the crypt of IDE. If true, the α-helical dichroic spectrum of insulin, showing two negative absorption bands centered at 222 and 208 nm and a positive absorption band centered below 200 nm [31], must change dramatically. Indeed, CD spectra of insulin in the presence of IDE recorded periodically show loss of the helical dichroic signals with increasing digestion time (Figure 2A). In sharp contrast, the dichroic spectrum of insulin did not change in the presence of E111Q IDE (Figure 2B), demonstrating that in the presence of the inactive form of IDE, insulin does not unfold.

To show unambiguously that the loss of helical content in Figure 2A was due to insulin degradation, we performed digestions using conditions similar to those used in preparing the CD samples in Figure 2 (i.e., 20 μM insulin in 50 mM Tris buffer (pH 7.4), substrate-to-enzyme molar ratio of 100:1, digestion temperature of 37°C). Aliquots of the digests were taken periodically for analysis by LC/MS. To unambiguously determine where the cleavages occur, insulin in the quenched digests was reacted with dithiothreitol to reduce Cys residues followed by alkylation using iodoacetamide to prevent the reformation of disulfide bonds. Supplementary Figure S1 presents the mass spectra of the 1-min, 3-h, and 24-h digests. The signals detected in the spectrum of the 1-min digest include peaks corresponding to intact A and B chains and peaks corresponding to two fragments of the A chain, Gly^A1^−Leu^A13^ and Tyr^A14^−Asn^A21^ (Supplementary Figure S1A, Supplementary Table S1). With an increase in digestion time to 3 h, peaks corresponding to additional fragments were detected along with peaks for intact A and B chains that were of lower intensities relative to the 1-min spectrum (Supplementary Figure S1B and Supplementary Table S1). At 24 h, no peak corresponding to intact A or B was detected, indicating complete degradation of insulin (Supplementary Figure S1C, Supplementary Table S1).

**Figure 3:**
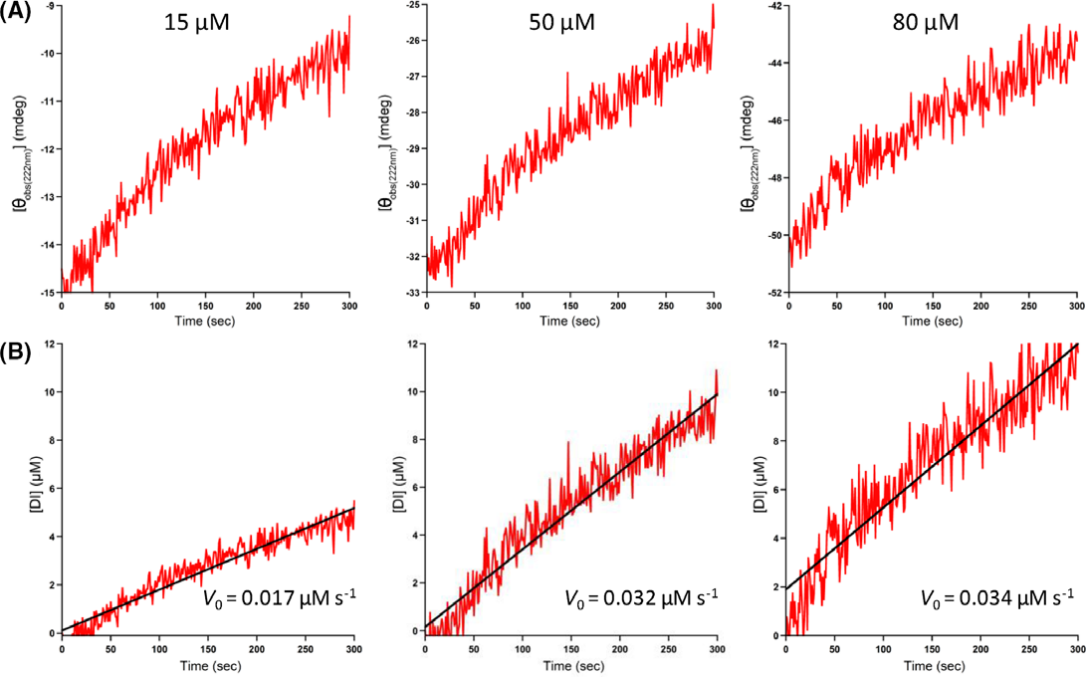
Early-stage kinetics of the IDE-dependent degradation of insulin in 50 mM Tris buffer (pH 7.4) at 37°C using observed ellipticity at 222 nm ([*θ*_obs(222 nm)_]) (**A**) Representative real-time plots of [*θ*_obs(222 nm)_] versus digestion time (0–300 s). The initial substrate concentration is indicated at the top of each plot. (**B**) Corresponding real-time plots of digested insulin ([DI]), calculated using eqn 1, versus digestion time. The R^2^ values of the fitting of the data to straight lines are (from left to right) 0.92, 0.92, and 0.88. The slope of each line yields *V_0_*.

**Figure 4:**
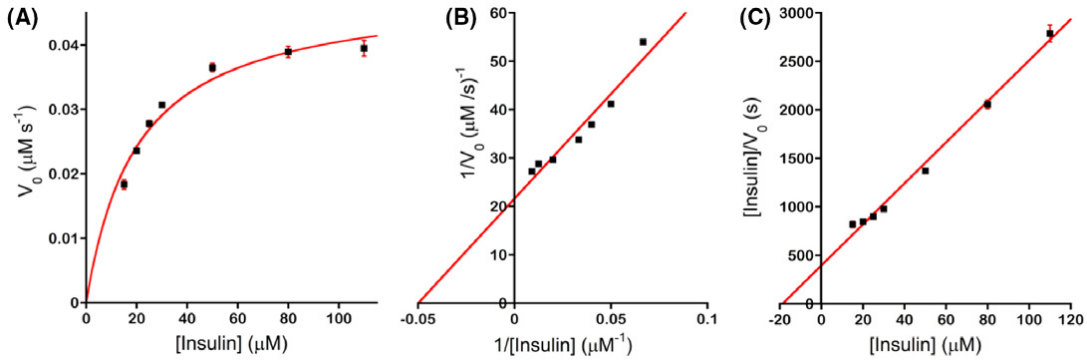
The IDE-dependent degradation of insulin at pH 7.4 and 37°C follows Michaelis–Menten kinetics Kinetic plots including (**A**) Michaelis–Menten, (**B**) Lineweaver–Burk, and (**C**) Hanes–Woolf plots were obtained from *V*_0_ determined from real-time plots of [*θ*_obs(222 nm)_] versus digestion time. Each solid square represents the mean of *V_0_*, 1/*V_0_*, or [insulin]/*V_0_* from three kinetic trials and the error bars represent standard deviations.

Next, we calculated the well-known Michaelis–Menten kinetic constants using the observed ellipticity at 222 nm ([*θ*_obs(222 nm)_]). After adding IDE to insulin, we recorded in real time [*θ*_obs(222 nm)_] and observed that the ellipticity increases (i.e., it becomes less negative indicating loss of helical structure [32]) with an increase in digestion time (Figure 3A). Based on the assumption that the increase in [*θ*_obs(222 nm)_] correlates with the amount of insulin digested, we thus used the following equation to approximate the amount of insulin digested over time:

(1) $${\left[\text{DI}\right]}_{t}={\left[\text{I}\right]}_{0}\times \left(1-\frac{{\left[{\text{θ}}_{\text{obs}(222\text{ nm})}\right]}_{t}}{{\left[{\text{θ}}_{\text{obs}(222\text{ nm})}\right]}_{0}}\right)$$

where [DI]*_t_* is the amount of insulin digested at time *t*, [I]_0_ is the initial amount of undigested insulin, and [*θ*_obs(222 nm)_]*_t_* and [*θ*_obs(222 nm)_]_0_ are the observed ellipticities at 222 nm at time *t* and time = 0, respectively. We noted that if [*θ*_obs(222 nm)_]*_t_* equals [*θ*_obs(222 nm)_]_0_, i.e., there is no change in the ellipticity, (eqn 1) correctly predicts that the amount of digested insulin is 0. Figure 3B presents representative plots of [DI]*_t_* against digestion time. Linear regression analysis yields *V*_0_, the initial velocity (or initial rate) of the IDE-catalyzed degradation of insulin. We used seven initial concentrations of the substrate ([S]) and kept the concentration of IDE constant and determined *V*_0_ from plots similar to Figure 3B. To obtain an initial proof of concept, we constructed Michaelis–Menten, Lineweaver–Burk and Hanes–Woolf plots (Figure 4), and obtained the expected hyperbolic increase in *V*_0_ with an increase in [S], linear increase in 1/*V*_0_ with an increase in 1/[S], and linear increase in [S]/*V*_0_ with an increase in [S], respectively. From these plots, we determined the steady-state kinetic parameters *K*_M_, *k*_cat_, and *k*_cat_/*K*_M_. Table 1 presents the parameters determined from the Michaelis–Menten plots. Similar results were obtained from the Lineweaver–Burk (Supplementary Table S2) and Hanes–Woolf (Supplementary Table S3) plots. We noted that the specificity constant *k*_cat_/*K*_M_ of 2.4 × 10^3^ M^−1^ s^−1^ (Table 1) is less than the value of 4.0 × 10^4^ M^−1^ s^−1^ determined by Chesneau and Rosner for recombinant human IDE and ^125^I-insulin (iodinated at Tyr^A14^) [33]. We speculate that the higher *k*_cat_/*K*_M_ obtained by Chesneau and Rosner is due to an enhancement of the interaction of chain A of insulin with the active site of IDE, resulting in an increased rate of cleavage of the peptide bond between Leu^A13^ and Tyr^A14^, the bond that is cleaved initially by IDE as determined by limited proteolysis (LP) (*vide infra*).

**Table 1:** Steady-state kinetic parameters^1^ for the degradation of insulin by IDE at pH 7.4 and 37°C determined from Michaelis–Menten plots

Regulator	*K*_M_ (M)	*k*_cat_ (s^−1^)	*k*_cat_/*K*_M_ (M^−1^s^−1^)
None	2.0 ± 0.04 × 10^−5^	0.048 ± 0.08	2.4 ± 0.02 × 10^3^
1 mM ATP	3.0 ± 0.2 × 10^−5^	0.056 ± 0.2	1.8 ± 0.07 × 10^3^
1 mM ATP + 1 mM Mg^2+^	2.0 ± 0.05 × 10^−5^	0.044 ± 0.07	2.1 ± 0.02 × 10^3^

1 Values are the means ± S.D. from three trials.

To determine the effect of ATP on the IDE-dependent hydrolysis of insulin and to obtain additional proof of concept, we repeated our digestion experiments in the presence of 1 mM ATP and 1 mM ATP plus 1 mM Mg^2+^. Table 1, Supplementary Tables S2 and S3 present the kinetic parameters determined from dichroic data similar to those shown in Figure 3A. Our results show that the addition of 1 mM ATP decreases *k*_cat_/*K*_M_ by 25 % (Table 1, Supplementary Tables S2 and S3), consistent with previous work by Song and co-workers showing that ATP regulates the activity of IDE toward large substrates including insulin [17]. The regulation may be mediated by the electrostatic interaction of the negatively charged triphosphate moiety of ATP with the positively charged interior of IDE-C [10,11]. Additionally, we found that when an equimolar amount of Mg^2+^ is present, ATP has no effect on *k*_cat_/*K*_M_ (Table 1, Supplementary Tables S2 and S3), suggesting that the presence of Mg^2+^ modulates the electrostatic interaction between ATP and IDE-C. Together, our results provide strong proof of concept for the validity of our CD-based assay.

**Figure 5:**
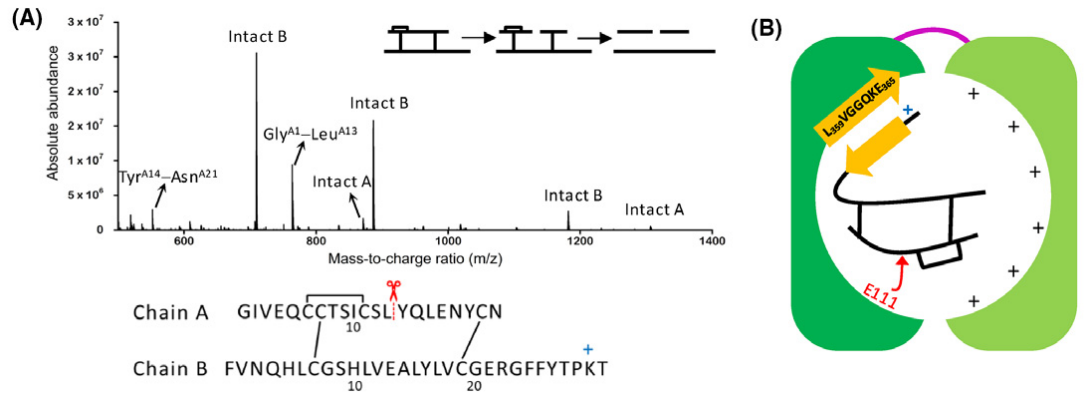
Limited proteolysis of insulin by IDE Insulin (100 μM in 50 mM Tris buffer (pH 7.4) was digested at 4°C by IDE using a substrate to enzyme molar ratio of 100:1. Quenched digests were reduced and alkylated for the unambiguous identification of the initial cleavage site by mass spectrometry. (**A**) Mass spectrum of the 1-min digest. The peaks corresponding to intact A, intact B, Gly^A1^−Leu^A13^, and Tyr^A14^−Asn^A21^ are identified. The inset shows schematically how the reduction and alkylation of insulin make possible the unambiguous identification of the initial cleavage site. Below the mass spectrum, the peptide map of insulin shows that the site of the initial cleavage is the peptide bond between Leu^A13^ and Tyr^A14^. (**B**) Schematic representation of the orientation of insulin in the crypt of IDE. IDE-N and IDE-C are shown in dark green and light green, respectively. We hypothesize that the C-terminal segment of chain B of insulin interacts with one of the β-strands in IDE’s exosite, mediated in part by the electrostatic interaction between the positively charged lysine residue in the C-terminus of the B chain of insulin (Lys^B29^), and the negatively charged glutamate residue in the exosite of IDE (E365), directing the initial cleavage to take place near the middle of chain A.

We noted that the *k*_cat_ of IDE in the absence or presence of ATP is more than two orders of magnitude slower than the median turnover of ∼10 s^−1^ determined by Bar-Even and co-workers from an analysis of several thousand enzymes and their natural substrates [34], let alone compared with *k*_cat_ of well-known examples of fast enzymes (e.g., superoxide dismutase and carbonic anhydrase), or to the theoretical limit of 10^6^–10^7^s^−1^ [35]. The *k*_cat_/*K*_M_ of IDE in the absence and presence of ATP is two orders of magnitude smaller than the median *k*_cat_/*K*_M_ of ∼10^5^ M^−1^s^−1^ determined by Bar-Even and co-workers [34]. Together, these observations suggest that IDE, when compared with other enzymes in the literature, is inefficient in degrading insulin. To explain the low *k*_cat_ and *k*_cat_/*K*_M_ of IDE, we noted that it has been hypothesized that IDE is specific to β-structure-forming substrates [1,2,6], presumably because the substrate forms β-sheet-like interactions with IDE prior to degradation [36]. To determine the orientation of insulin in the crypt of IDE during proteolysis, we used LP together with LC/MS. We have previously used LP and LC/MS to successfully elucidate the conformation of monomeric states of amyloidogenic peptides including Aβ [37,38] and amylin [39]. By applying the conditions for LP including substrate-to-enzyme molar ratios ≥100:1, digestion temperature of 4°C, and short digestion times (∼1 min), the initial cleavage site in insulin can be identified and then used as a constraint in deciphering the arrangement of insulin monomer in the crypt of IDE. Figure 5A presents the mass spectrum of the 1-min digest. Interestingly, the spectrum is similar to the mass spectrum of the 1-min digest at 37°C (Supplementary Figure S1A) in that the peaks detected in the spectrum include peaks corresponding to intact A and B chains and peaks corresponding to fragments of the A chain including Gly^A1^−Leu^A13^ and Tyr^A14^−Asn^A21^. This result unambiguously indicates that the initial cleavage occurs in chain A of insulin, particularly at the peptide bond between Leu^A13^ and Tyr^A14^. This agrees with the current working model of how IDE sequentially cleaves insulin [8] and with studies of the IDE-dependent hydrolysis of insulin by LC/MS [40] and by matrix assisted laser desorption ionization mass spectrometry [41]. X-ray structures of IDE show that its exosite forms an anti-parallel β-sheet (Figure 1A) consisting of a β-strand (L_359_VGGQKE_365_), a β-turn (G_366_ARG_369_), and a second β-strand (F_370_FFIINV_376_) [7]. The underlined residues are found in the crypt of IDE. The only way for the initial cleavage to occur in chain A of insulin is for the exosite of IDE to bind the C-terminal segment of the B chain of insulin (Figure 5B), not its N-terminus, as previously hypothesized [1,8]. We noted that the C-terminal segment of the B chain of insulin has a high propensity to form β-sheet in that in the structure of insulin dimer [31], the repeating unit in the hexameric form of the protein *in vivo*, the C-terminal segment of the B chain of one monomer forms a β-sheet with the C-terminal segment of the B chain of the other monomer. We speculate that the anchoring of the C-terminal segment of the B chain of insulin to IDE’s exosite is facilitated by interactions of the (L_359_VGGQKE_365_) β-strand of IDE with the C-terminal segment of insulin, including an electrostatic attraction between Lys^B29^ and E365, thereby directing the initial cleavage to occur in the unstructured middle part of the A chain (Figure 5B). Our model supports the hypothesis that the specificity of IDE to β-structure-forming susbstrates is due to the interaction of the non-helical part of the substrate with the β-sheet structure of IDE’s highly conserved exosite. IDE is slow and inefficient in its turnover of insulin because this substrate is predominantly α-helical.

How might our results summarized in Table 1 be exploited in the development of therapeutic and/or preventative strategies for AD? Recently, Patel and co-workers showed that ATP at physiological concentrations in the presence of an equimolar amount of Mg^2+^ is a biological hydrotrope in that it increases the solubility of proteins and prevents the aggregation of monomers of Aβ42 [42], one of the most amyloidogenic forms of Aβ [43]. This finding suggests that the ability of IDE to degrade monomeric Aβ42 may be enhanced in the presence of ATP. Because levels of ATP decline with ageing or mitochondrial impairment [44,45], our results as summarized in Table 1, suggest that preventative strategies aimed at maintaining the normal levels of ATP and Mg^2+^ may boost the IDE-dependent clearance of Aβ42 without offsetting the normal levels of insulin.

In conclusion, we have presented a novel assay for the determination of Michaelis–Menten kinetic constants for the IDE-dependent degradation of unlabeled insulin. We anticipate that this assay will inform us of other small molecules that regulate the degradation of insulin. A full understanding of the IDE-dependent degradation of insulin is key in the development of therapeutic strategies targetting IDE to treat T2D and AD.

## Supporting information

**Figure S1:** Proteolysis of insulin by IDE at 37°C. Insulin (100 μM in 50 mM Tris buffer, pH 7.4) was digested by IDE using a substrate-to-enzyme molar ratio of 100:1. Quenched digests were reduced and alkylated for the unambiguous identification of products by mass spectrometry. (A) Mass spectrum of the 1-minute digest. Peaks corresponding to intact A, intact B and fragments due to the cleavage of the peptide bond between Leu^A13^ and Tyr^A14^ were detected. (B) Mass spectrum of the 3-hour digest. Peaks corresponding to intact A, intact B, and short fragments of the A and B chains were detected. (C) Mass spectrum of the 24-hour digest. No peak corresponding to intact A or intact B was observed indicating complete digestion of insulin.

**Table S1:** Chains and dominant fragments detected in the mass spectra of insulin digests.*

**Table S2:** Steady-state kinetic parameters for the degradation of insulin by IDE at pH 7.4 and 37°C determined from Lineweaver-Burk plots.

**Table S3:** Steady-state kinetic parameters for the degradation of insulin by IDE at pH 7.4 and 37°C determined from Hanes-Woolf plots.

## Abbreviations

Aβ: amyloid-β
AD: Alzheimer’s disease
ATP: adenosine triphosphate
CD: circular dichroism
FPLC: fast protein liquid chromatography
GST-IDE: glutathione-S-transferase tagged human insulin-degrading enzyme
IDE: insulin-degrading enzyme
IDE-C: C-terminal half of IDE
IDE-N: N-terminal half of IDE
LP: limited proteolysis
TCA: trichloroacetic acid
T2D: type 2 diabetes
UPLC: ultra performance liquid chromatography

## References

[1] Tang W.J. (2016) Targeting insulin-degrading enzyme to treat type 2 diabetes mellitus. Trends Endocrinol. Metab. 27, 24–34 10.1016/j.tem.2015.11.003 26651592 PMC4698235

[2] Kurochkin I.V., Guarnera E. and Berezovsky I.N. (2018) Insulin-degrading enzyme in the fight against Alzheimer’s disease. Trends Pharmacol. Sci. 39, 49–58 10.1016/j.tips.2017.10.008 29132916

[3] Lorenzo A., Razzaboni B., Weir G.C. and Yankner B.A. (1994) Pancreatic islet cell toxicity of amylin associated with type-2 diabetes mellitus. Nature 368, 756–760 10.1038/368756a0 8152488

[4] Saido T. and Leissring M.A. (2012) Proteolytic degradation of amyloid β-protein. Cold Spring Harb. Perspect. Med. 2, a006379 10.1101/cshperspect.a006379 22675659 PMC3367539

[5] Cline E.N., Bicca M.A., Viola K.L. and Klein W.L. (2018) The amyloid-β oligomer hypothesis: Beginning of the third decade. J. Alzheimer’s Dis. 64, S567–S610 10.3233/JAD-179941 29843241 PMC6004937

[6] Shen Y., Joachimiak A., Rosner M.R. and Tang W.J. (2006) Structures of human insulin-degrading enzyme reveal a new substrate recognition mechanism. Nature 443, 870–874 10.1038/nature05143 17051221 PMC3366509

[7] Durham T.B., Toth J.L., Klimkowski V.J., Cao J.X., Siesky A.M., Alexander-Chacko J. (2015) Dual exosite-binding inhibitors of insulin-degrading enzyme challenge its role as the primary mediator of insulin clearance *in vivo*. J. Biol. Chem. 290, 20044–20059 10.1074/jbc.M115.638205 26085101 PMC4536412

[8] Manolopoulou M., Guo Q., Malito E., Schilling A.B. and Tang W.J. (2009) Molecular basis of catalytic chamber-assisted unfolding and cleavage of human insulin by human insulin-degrading enzyme. J. Biol. Chem. 284, 14177–14188 10.1074/jbc.M900068200 19321446 PMC2682866

[9] McCord L.A., Liang W.G., Dowdell E., Kalas V., Hoey R.J., Koide A. (2013) Conformational states and recognition of amyloidogenic peptides of human insulin-degrading enzyme. Proc. Natl. Acad. Sci. U.S.A. 110, 13827–13832 10.1073/pnas.1304575110 23922390 PMC3752249

[10] Im H., Manolopoulou M., Malito E., Shen Y., Zhao J., Neant-Fery M. (2007) Structure of substrate-free human insulin-degrading enzyme (IDE) and biophysical analysis of ATP-induced conformational switch of IDE. J. Biol. Chem. 282, 25453–25463 10.1074/jbc.M701590200 17613531

[11] Noinaj N., Song E.S., Bhasin S., Alper B.J., Schmidt W.K., Hersh L.B. (2012) Anion activation site of insulin-degrading enzyme. J. Biol. Chem. 287, 48–57 10.1074/jbc.M111.264614 22049080 PMC3249101

[12] Guo Q., Manolopoulou M., Bian Y., Schilling A.B. and Tang W.J. (2010) Molecular basis for the recognition and cleavages of IGF-II, TGF-α, and amylin by human insulin-degrading enzyme. J. Mol. Biol. 395, 430–443 10.1016/j.jmb.2009.10.072 19896952 PMC2813390

[13] Perlman R.K., Gehm B.D., Kuo W.L. and Rosner M.R. (1993) Functional analysis of conserved residues in the active site of insulin-degrading enzyme. J. Biol. Chem. 268, 21538–21544 8104941

[14] Duckworth W.C., Bennett R.G. and Hamel F.G. (1998) Insulin degradation: progress and potential. Endocr. Rev. 19, 608–624 9793760 10.1210/edrv.19.5.0349

[15] Farris W., Mansourian S., Chang Y., Lindsley L., Eckman E.A., Frosch M.P. (2003) Insulin-degrading enzyme regulates the levels of insulin, amyloid β-protein, and the β-amyloid precursor protein intracellular domain *in vivo*. Proc. Natl. Acad. Sci. U.S.A. 100, 4162–4167 10.1073/pnas.0230450100 12634421 PMC153065

[16] Camberos M.C., Perez A.A., Udrisar D.P., Wanderley M.I. and Cresto J.C. (2001) ATP inhibits insulin-degrading enzyme activity. Exp. Biol. Med. (Maywood) 226, 334–341 10.1177/153537020122600411 11368426

[17] Song E.S., Juliano M.A., Juliano L., Fried M.G., Wagner S.L. and Hersh L.B. (2004) ATP effects on insulin-degrading enzyme are mediated primarily through its triphosphate moiety. J. Biol. Chem. 279, 54216–54220 10.1074/jbc.M411177200 15494400

[18] Duckworth W.C. (1990) Insulin-degrading enzyme. In Handbook of Experimental Pharmacology, (Cuatrecasas P. and Jacobs S., eds), pp. 143–165, Springer-Verlag

[19] El Hage K., Pandyarajan V., Phillips N.B., Smith B.J., Menting J.G. and Whittaker J (2016) Extending halogen-based medicinal chemistry to proteins: Iodo-insulin as a case study. J. Biol. Chem. 291, 27023–27041 10.1074/jbc.M116.761015 27875310 PMC5207135

[20] Lommerse J.P.M., Stone A.J., Taylor R.A. and Allen F.H. (1996) The nature and geometry of intermolecular interactions between halogens and oxygen or nitrogen. J. Am. Chem. Soc. 118, 3108–3116

[21] Metrangolo P., Neukirch H., Pilati T. and Resnati G. (2005) Halogen bonding based recognition processes: a world parallel to hydrogen bonding. Acc. Chem. Res. 38, 386–395 10.1021/ja953281x 10.1021/ja953281x 15895976

[22] Politzer P., Murray J.S. and Clark T. (2013) Halogen bonding and other σ-hole interactions: a perspective. Phys. Chem. Chem. Phys. 15, 11178–11189 10.1039/c3cp00054k 23450152

[23] Linde S., Sonne O., Hansen B. and Gliemann J. (1981) Monoiodoinsulin labelled in tyrosine residue 16 or 26 of the insulin B-chain. Preparation and characterization of some binding properties. Hoppe. Seylers Z. Physiol. Chem. 362, 573–579 10.1515/bchm2.1981.362.1.573 7024083

[24] Burant C.F., Treutelaar M.K., Peavy D.E., Frank B.H. and Buse M.G. (1988) Differential binding of monoiodinated insulins to muscle and liver derived receptors and activation of the receptor kinase. Biochem. Biophys. Res. Commun. 152, 1353–1360 10.1016/S0006-291X(88)80434-0 2837184

[25] Pandyarajan V., Phillips N.B., Cox G.P., Yang Y., Whittaker J., Ismail-Beigi F. (2014) Biophysical optimization of a therapeutic protein by nonstandard mutagenesis: Studies of an iodo-insulin derivative. J. Biol. Chem. 289, 23367–23381 10.1074/jbc.M114.588277 24993826 PMC4156050

[26] Noinaj N., Bhasin S.K., Song E.S., Scoggin K.E., Juliano M.A., Juliano L. (2011) Identification of the allosteric regulatory site of insulysin. PLoS ONE 6, e20864 10.1371/journal.pone.0020864 21731629 PMC3123307

[27] da Cruz C.H Seabra G. (2014) Molecular dynamics simulations reveal a novel mechanism for ATP inhibition of insulin degrading enzyme. J. Chem. Inf. Model. 54, 1380–1390 10.1021/ci400695m 24697863

[28] Sharma S.K., Chorell E., Steneberg P., Vernersson-Lindahl E., Edlund H. and Wittung-Stafshede P. (2015) Insulin-degrading enzyme prevents α-synuclein fibril formation in a nonproteolytical manner. Sci. Rep. 5, 12531 10.1038/srep12531 26228656 PMC4521159

[29] Sorci M., Grassucci R.A., Hahn I., Frank J. and Belfort G. (2009) Time-dependent insulin oligomer reaction pathway prior to fibril formation: cooling and seeding. Proteins 77, 62–73 10.1002/prot.22417 19408310 PMC2737737

[30] Gerstein A.S. (2001) Nucleotides, oligonucleotides, and polynucleotides. In Molecular Biology Problem Solver: A Laboratory Guide (Gerstein A.S., ed.), pp. 267–289, Wiley-Liss, Inc

[31] Zheng Q. and Lazo N.D. (2018) Mechanistic studies of the inhibition of insulin fibril formation by rosmarinic acid. J. Phys. Chem. B 122, 2323–2331 10.1021/acs.jpcb.8b00689 29401384

[32] Lazo N.D. and Downing D.T. (1997) Circular dichroism of model peptides emulating the amphipathic α-helical regions of intermediate filaments. Biochemistry 36, 2559–2565 10.1021/bi963061b 9054562

[33] Chesneau V. and Rosner M.R. (2000) Functional human insulin-degrading enzyme can be expressed in bacteria. Protein Expr. Purif. 19, 91–98 10.1006/prep.2000.1217 10833395

[34] Bar-Even A., Noor E., Savir Y., Liebermeister W., Davidi D., Tawfik D.S. (2011) The moderately efficient enzyme: evolutionary and physicochemical trends shaping enzyme parameters. Biochemistry 50, 4402–4410 10.1021/bi2002289 21506553

[35] Hammes G.G. (2002) Multiple conformational changes in enzyme catalysis. Biochemistry 41, 8221–8228 10.1021/bi0260839 12081470

[36] Krasinski C.A., Zheng Q., Ivancic V.A., Spratt D.E. and Lazo N.D. (2018) The longest amyloid-β precursor protein intracellular domain produced with Aβ42 forms β-sheet-containing monomers that self-assemble and are proteolyzed by insulin-degrading enzyme. ACS Chem. Neurosci., 10.1021/acschemneuro.8b00305 PMC7074877 30067897

[37] Lazo N.D., Grant M.A., Condron M.C., Rigby A.C. and Teplow D.B. (2005) On the nucleation of amyloid β-protein monomer folding. Protein Sci. 14, 1581–1596 10.1021/acschemneuro.8b00305 10.1021/acschemneuro.8b00305 15930005 PMC2253382

[38] Grant M.A., Lazo N.D., Lomakin A., Condron M.M., Arai H., Yamin G. (2007) Familial Alzheimer’s disease mutations alter the stability of the amyloid β-protein monomer folding nucleus. Proc. Natl. Acad. Sci. U.S.A. 104, 16522–16527 10.1073/pnas.0705197104 17940047 PMC2034231

[39] Liu G., Prabhakar A., Aucoin D., Simon M., Sparks S., Robbins K.J. (2010) Mechanistic studies of peptide self-assembly: Transient α-helices to stable β-sheets. J. Am. Chem. Soc. 132, 18223–18232 10.1021/ja1069882 21138275

[40] Bellia F., Pietropaolo A. and Grasso G. (2013) Formation of insulin fragments by insulin-degrading enzyme: the role of zinc(II) and cystine bridges. J. Mass Spectrom. 48, 135–140 10.1002/jms.3060 23378084

[41] Grasso G., Rizzarelli E. and Spoto G. (2009) The proteolytic activity of insulin-degrading enzyme: a mass spectrometry study. J. Mass Spectrom. 44, 735–741 10.1002/jms.1550 19127548

[42] Patel A., Malinovska L., Saha S., Wang J., Alberti S., Krishnan Y. (2017) ATP as a biological hydrotrope. Science 356, 753–756 10.1126/science.aaf6846 28522535

[43] Lazo N.D., Maji S.K., Fradinger E.A., Bitan G. and Teplow D.B. (2005) The amyloid-β protein. In Amyloid Proteins: The Beta Sheet Conformation and Disease (Sipe J.C., ed.), pp. 385–491, Wiley-VCH

[44] Swerdlow R.H. (2011) Brain aging, Alzheimer’s disease, and mitochondria. Biochim. Biophys. Acta 1812, 1630–1639 10.1016/j.bbadis.2011.08.012 21920438 PMC3210037

[45] Wang X., Su B., Lee H.G., Li X., Perry G., Smith M.A. (2009) Impaired balance of mitochondrial fission and fusion in Alzheimer’s disease. J. Neurosci. 29, 9090–9103 10.1523/JNEUROSCI.1357-09.2009 19605646 PMC2735241

