# Supplementary material for: Enzyme kinetics from circular dichroism of insulin reveals mechanistic insights into the regulation of insulin-degrading enzyme
Source: Biosci Rep. 2018 Nov 7;38(6):BSR20181416. doi: 10.1042/BSR20181416 (PMC6239264; doi:10.1042/BSR20181416)
Supplement: Supplementary file 1 [file bsr20181416_Supp1.pdf]

Supporting Information for

Enzyme Kinetics from Circular Dichroism of Insulin Reveals Mechanistic Insights into the Regulation of Insulin-degrading Enzyme

Valerie A. Ivancic, Claire A. Krasinski, Qiuchen Zheng, Rebecca J. Meservier,
Donald E. Spratt, and Noel D. Lazo*

Carlson School of Chemistry and Biochemistry, Clark University, 950 Main Street, Worcester, Massachusetts 01610, United States

*nlazo@clarku.edu

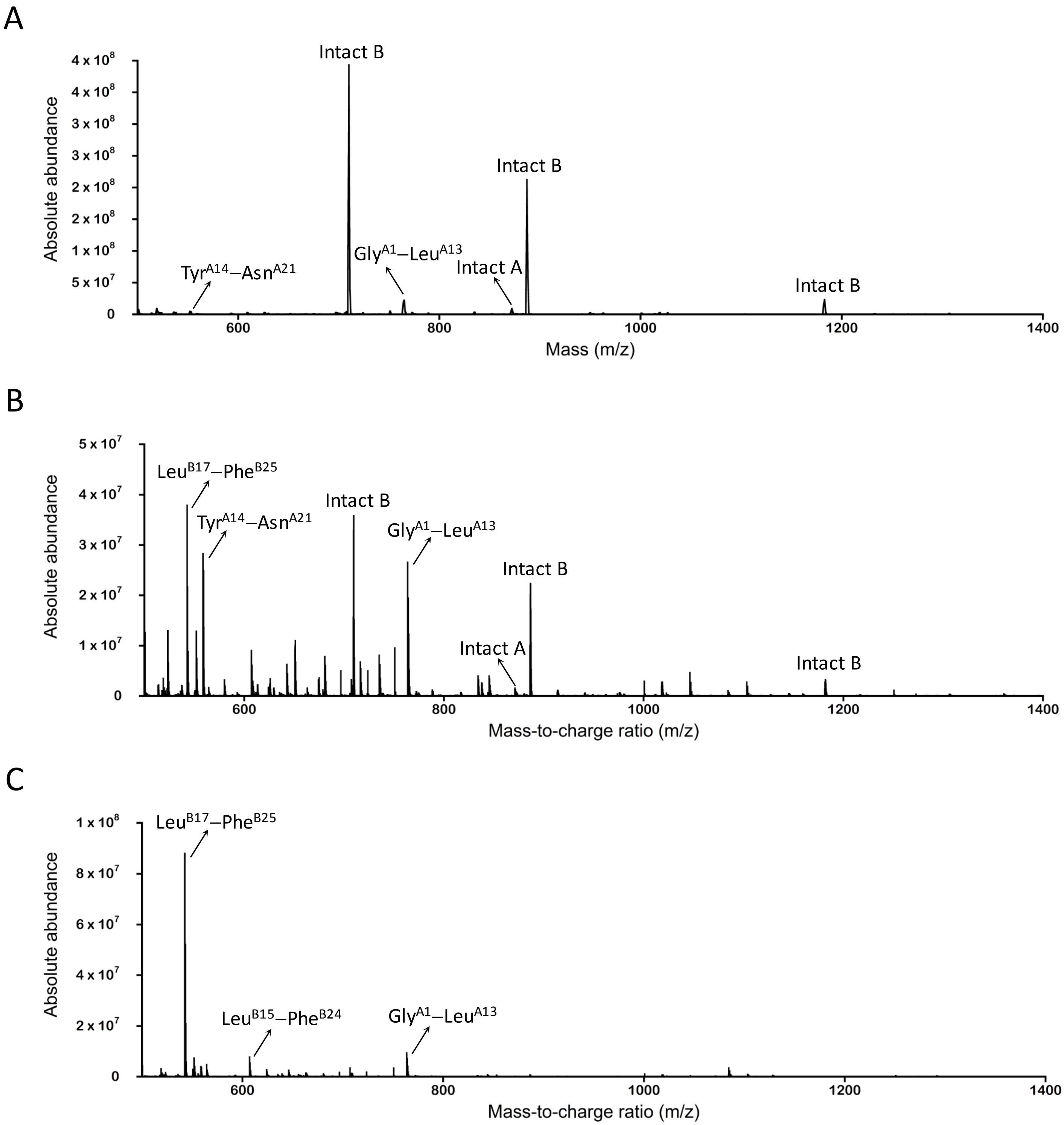

Figure S1. Proteolysis of insulin by IDE at 37 °C. Insulin (100 μM in 50 mM Tris buffer, pH 7.4) was digested by IDE using a substrate-to-enzyme molar ratio of 100:1. Quenched digests were reduced and alkylated for the unambiguous identification of products by mass spectrometry. (A) Mass spectrum of the 1-minute digest. Peaks corresponding to intact A, intact B and fragments due to the cleavage of the peptide bond between Leu^{A13} and Tyr^{A14} were detected. (B) Mass spectrum of the 3-hour digest. Peaks corresponding to intact A, intact B, and short fragments of the A and B chains were detected. (C) Mass spectrum of the 24-hour digest. No peak corresponding to intact A or intact B was observed indicating complete digestion of insulin.

Table S1. Chains and dominant fragments detected in the mass spectra of insulin digests.*

Chain or Fragment	Observed Mass (Da)	Theoretical Mass (Da)	δ** (Da)
**A. 1-minute digestion**			
Intact A (Gly^{A1}–Asn^{A21})	2382.12	2382.00	0.12
Intact B (Phe^{B1}–Thr^{B30})	3427.80	3427.69	0.11
Gly^{A1}–Leu^{A13}	1354.68	1354.60	0.08
Tyr^{A14}–Asn^{A21}	1045.46	1045.42	0.04
**B. 3-hour digestion**			
Intact A (Gly^{A1}–Asn^{A21})	2382.12	2382.00	0.12
Intact B (Phe^{B1}–Thr^{B30})	3427.80	3427.69	0.11
Gly^{A1}–Ser^{A12}	1241.58	1241.51	0.07
Leu^{A13}–Asn^{A21}	1158.54	1158.50	0.04
Gly^{A1}–Leu^{A13}	1354.68	1354.60	0.08
Tyr^{A14}–Asn^{A21}	1045.46	1045.42	0.04
Phe^{B1}–Ser^{B9}	1003.50	1003.46	0.04
His^{B10}–Thr^{B30}	2442.27	2442.24	0.03
Phe^{B1}–His^{B10}	1140.57	1140.52	0.05
Leu^{B11}–Thr^{B30}	2305.23	2305.18	0.05
Phe^{B1}–Glu^{B13}	1481.76	1481.71	0.05
Ala^{B14}–Thr^{B30}	1964.02	1963.99	0.03
Phe^{B1}–Ala^{B14}	1552.80	1552.75	0.05
Leu^{B15}–Thr^{B30}	1892.98	1892.95	0.03
Phe^{B1}–Leu^{B15}	1665.86	1665.83	0.03
Tyr^{B16}–Thr^{B30}	1779.90	1779.87	0.03
Phe^{B1}–Tyr^{B16}	1828.95	1828.90	0.05
Leu^{B17}–Thr^{B30}	1616.84	1616.80	0.04
Phe^{B1}–Gly^{B20}	2201.14	2201.08	0.06
Glu^{B21}–Thr^{B30}	1244.64	1244.62	0.02
Phe^{B1}–Gly^{B23}	2543.32	2543.24	0.08
Phe^{B24}–Thr^{B30}	902.46	902.46	0.00
**C. 24-hour digestion**			
Gly^{A1}–Leu^{A13}	1354.68	1354.60	0.08
Leu^{B17}–Phe^{B25}	1026.54	1027.21	-0.67
Leu^{B15}–Phe^{B24}	1155.62	1156.36	-0.74

* 100 µM insulin in 50 mM Tris buffer, pH 7.4, 37 °C, substrate to enzyme molar ratio of 100:1
**δ = Observed Mass – Theoretical Mass

Table S2. Steady-state kinetic parameters for the degradation of insulin by IDE at pH 7.4 and 37 °C determined from Lineweaver-Burk plots.

Regulator	K_M (M)	k_{cat} (s^{-1})	k_{cat}/K_M ($M^{-1}s^{-1}$)
None	$2.7 \pm 0.16 \times 10^{-5}$	0.054 ± 0.001	$2.0 \pm 0.08 \times 10^{3}$
1 mM ATP	$3.8 \pm 0.18 \times 10^{-5}$	0.062 ± 0.001	$1.6 \pm 0.06 \times 10^{3}$
1 mM ATP + 1 mM Mg^{2+}	$2.4 \pm 0.05 \times 10^{-5}$	0.046 ± 0.002	$1.9 \pm 0.09 \times 10^{3}$

[1]Values are the means ± SD from three trials.

Table S3. Steady-state kinetic parameters for the degradation of insulin by IDE at pH 7.4 and 37 °C determined from Hanes-Woolf plots.

Regulator	K_M (M)	k_{cat} (s^{-1})	k_{cat}/K_M ($M^{-1}s^{-1}$)
None	$1.9 \pm 0.09 \times 10^{-5}$	0.047 ± 0.001	$2.5 \pm 0.07 \times 10^{3}$
1 mM ATP	$3.1 \pm 0.2 \times 10^{-5}$	0.056 ± 0.002	$1.8 \pm 0.08 \times 10^{3}$
1 mM ATP + 1 mM Mg^{2+}	$2.1 \pm 0.09 \times 10^{-5}$	0.044 ± 0.001	$2.1 \pm 0.06 \times 10^{3}$

[1]Values are the means ± SD from three trials.
